# How Glands Influence Character: A New Conception in Physiology

**Published:** 1924-06

**Authors:** 


### HOW GLANDS INFLUENCE DEVELOPMENT.

### A NEW CONCEPTION IN PHYSIOLOGY.

Lecturing at Sheffield in the series of addresses on "Health Education" upon the "Effect of Medical Research on Public Welfare," Dr. C. J. Bond, C.M.G., F.R.C.S., Vice-chairman of the Medical Consultative Council, Ministry of Health, said that he wanted to engage attention in regard to certain little organs in the body about which not much was known but which medical research had shown to be of outstanding importance to the individual. He referred to the pituitary gland, which, by means of a diagram on the screen, he showed to be located at the base of the brain, the thyroid gland situated in the neck, and the supra-renal gland which formed a little cap on the top of each kidney. It was on the normal working of the pituitary gland that it depended whether a person was normal in stature, or a giant or a dwarf. Sometimes, owing to the early ossification of the cartilage of the ends of the bones, and this gland not working properly, a person became stunted in growth. It was very interesting to note that dwarfs existed 2,700 years before Christ, in proof of which Dr. Bond showed a picture of a statue of such a dwarf who was called "The Master of the Perfumes," and was a Court Chamberlain of Pharaoh's household. The racial dwarfs found in the Congo Region, on the other hand, did not owe their diminutive stature to abnormal conditions of the pituitary gland.

### Gland Secretions.

Passing on to the thyroid gland, Dr. Bond explained that failure of proper function on the part of this gland caused enlarged neck, or goitre—a common affection in Switzerland, Derbyshire and other parts of the world. It was now known that if during infancy the gland failed, or its secretion was insufficient, then the child suffered from arrested or abnormal growth, and the skin and hair suffered considerably. If, later in life, the gland should become disordered there followed a corresponding interference with the normal nutrition of the body, loss of hair, etc. The value of research was shown in the fact that it had been discovered that the trouble could be relieved by taking sheep's thyroid gland in tabloid form. The supra-renal gland had also a great bearing on the secretion of the sex glands. They would all realise how at the age of puberty boys and girls developed different characteristics, and these mental and bodily attributes were due to the internal secretion of the sex glands acting on the brain and bodily tissues. He wanted everybody to realise the importance of this dual function of the sex glands.

### The Activities of the Body.

A new conception had arisen in physiology. Just as they had formerly regarded the nervous system as the co-ordinating agent, so they were coming to see through the labours of Professor Starling and other workers that, besides this nervous co-ordination, the body depended on a system of internal secretions. The nervous system might be regarded as the "telegraphic" co-ordinating system of the body. Messages were sent through to the central nervous organs, and messages directing action and secretions were sent out. But in addition to the "telegraphic" system, there was also a system of regulation or co-ordination of the body by means of a "postal" system. Messages or secretions from the organs he had mentioned were carried in the blood stream to the tissues where they were required. By means of the "telegraphic" system and the "postal" system the whole co-ordination of the activities of the human body was carried on.

### Why We Should Eat Brown Bread.

Certain substances in the food were necessary for normal growth and nutrition of the body. The first thing to note in connection with vitamins was that at present they had not been obtained in a chemically pure state; they influenced the growth of the body in youth and the nutrition of the body throughout life. They are found in three main groups. The first of these is the anti-scorbutic, or anti-scurvy, group. These are soluble in water, are present in vegetables, fruit and green foods, and to a less extent in milk, eggs and animal food. The second group is known as the anti-neuritic group. This vitamin is found in the outer coats of cereals such as rice, oats and wheat. He wanted to mention in connection with this the story of the Norwegian skipper who had been travelling to the Arctic regions for many years, and always took with him a supply of brown flour. Members of his crew, having mixed with other crews, felt that they ought to have white bread and not the "inferior" brown bread. The skipper knew better, and for his own private consumption took brown flour. When they had been sailing in the Arctic for some time, the members of the crew began to suffer owing to their use of white flour. By carefully rationing out his brown flour, the skipper not only kept well himself but managed to restore his crew to a fairly good state of health. In the present day in England, the so-called offal of cereals, bran, etc., fed to animals is worth more, said Dr. Bond, than the wheat flour we are eating.

### Heredity and Disease.

In conclusion, Dr. Bond showed examples of cross-fertilisation in plants, fowls and doves, and in the human species, and from this advanced the conclusion that heredity played a great part not only in disease, but in the development and welfare of the human race. He quoted instances of certain long-lived families, and also of some with defects, such as "thumb-fingeredness," and went on to point out the danger of allowing those who were known to be feeble-minded to perpetuate their defect. He urged the necessity that for the good of the nation such defectives should be segregated in colonies for life. He added that the race that would have the courage to look after its child life and to eradicate these defective strains would be the race to inherit the earth in the future.

